# The effectiveness of volunteer befriending for improving the quality of life of patients with schizophrenia in Bosnia and Herzegovina – an exploratory randomised controlled trial

**DOI:** 10.1017/S2045796021000330

**Published:** 2021-06-11

**Authors:** H. Sikira, S. Janković, Murga S. Slatina, M. Muhić, S. Sajun, S. Priebe, A. Džubur Kulenović

**Affiliations:** 1 Department of Psychiatry, Clinical Centre University of Sarajevo, Bosnia, Herzegovina; 2 Faculty of Medical Sciences, University of Kragujevac, Kragujevac, Serbia; 3 Unit for Social and Community Psychiatry, WHO Collaborating Centre for Mental Health Services Development, Queen Mary University of London, London, UK

**Keywords:** Befriending, community mental health, psychosis, social isolation, volunteers

## Abstract

**Aims:**

Social isolation in people living with schizophrenia is associated with poor quality of life and increased symptom severity. Volunteer befriending interventions are a potential strategy for addressing social isolation, but evidence of their effectiveness is limited, particularly in low- and middle-income countries. We assessed the experiences of volunteer befriending and tested its effectiveness for improving the quality of life of patients with schizophrenia in Bosnia and Herzegovina.

**Methods:**

Between March 2018 and July 2020, we conducted a parallel-group, randomised controlled trial in adults with schizophrenia and poor quality of life at an outpatient clinic in Sarajevo. Patients were randomised to either the intervention, in which they were matched with a volunteer befriender with whom they met fortnightly over the 6-month intervention period, or treatment as usual. The primary outcome was quality of life measured on the Manchester Short Assessment and secondary outcomes were psychiatric symptoms and objective social outcomes. Outcome measurement was conducted by blinded researchers at 6- and 12-months.

**Results:**

In total, 65 patients were randomised into the intervention (*n* = 33) and control arms (*n* = 32) and 55 (85%) completed follow-up assessments at 6 months. Patients in the intervention showed a significantly more favourable quality of life at 6 months (primary outcome; mean difference: 0.7, 95% CI [0.3–1.1], *p* = 0.003) and 12 months (mean difference: 1.7, 95% CI [1.1–2.3], *p* < 0.001). They also had significantly lower symptom levels at both follow-ups, and a significantly more favourable objective social situation after 12 months. Participants reported largely positive experiences.

**Conclusion:**

The exploratory trial conducted at one site found sustained improvements in quality of life and reductions in psychiatric symptoms. This suggests that volunteer befriending may be a feasible and effective treatment for patients with schizophrenia in resource-limited contexts, such as Bosnia and Herzegovina.

## Introduction

People living with schizophrenia have smaller social networks and experience more social isolation than the general population (Harley *et al*., 2012; Giacco *et al*., 2016), which is associated with poorer quality of life, increased symptom severity and reduced treatment adherence (Becker *et al*., 1998; Giacco *et al*., 2012; Stentzel *et al*., 2018). The symptoms of schizophrenia can affect an individual's ability and interest in establishing social contact. Positive symptoms, such as hallucinations and delusions, are associated with active social avoidance, while negative symptoms reduce the motivation to build and maintain social relationships (Hansen *et al*., 2009; de Sousa *et al*., 2018). Poorer psychosocial outcomes in people living with schizophrenia, such as reduced employment opportunities, insecure housing, financial difficulties and diminished social role, further reduce social networks (Harley *et al*., 2012). At the same time, mental health stigma and prejudice towards people with severe mental illness compound experiences of social isolation (Angermeyer and Dietrich, 2006; Livingston and Boyd, 2010).

Volunteer befriending interventions are a potential resource for addressing social isolation. These interventions link volunteers with patients to provide social and practical support through regular one-to-one meetings (Mitchell and Pistrang, 2011; Thompson *et al*., 2016; Siette *et al*., 2017). While evidence to support the effectiveness of volunteer befriending is mixed (McCorkle *et al*., 2008; Mead *et al*., 2010), there is some evidence befriending patients with severe mental illness improves perceived social support, reduces social isolation and leads to a long-term increase in social contacts (Hallett *et al*., 2012; Sheridan *et al*., 2015; Priebe *et al*., 2020). Further, the willingness for members of the public to voluntarily spend time with people with severe mental illness presents an inexpensive resource for the design and delivery of mental health services (Priebe *et al*., 2014; Klug *et al*., 2018). As a low-cost therapeutic model that addresses resources rather than deficits, volunteer befriending interventions could be a viable option for low- and middle-income countries where mental health workforce is limited and health systems are fragmented, such as Bosnia and Herzegovina (Kucukalic *et al*., 2005; Fuhr *et al*., 2014).

Bosnia and Herzegovina suffered four years of continuous conflict leading to an increased prevalence of trauma-related psychiatric disorders. At the same time, many psychiatric institutions, including long-term treatment facilities, were physically destroyed over the course of the war. These two events led to a reform of mental health services since the conclusion of the conflict (Kucukalic *et al*., 2005). Economic transition and political instability have discouraged the return of refugees and encouraged economic migration, particularly of health professionals, with implications for the mental health workforce. As a result, there is a lack of services for people with severe mental illness beyond pharmacological prescription, such as psychosocial interventions (Winkler *et al*., 2017; Hunter *et al*., 2020).

We test the feasibility and effectiveness of a volunteer befriending intervention for improving quality of life for patients with schizophrenia or non-affective psychosis in Bosnia and Herzegovina.

## Methods

### Study design and participants

We conducted an exploratory parallel-group, pragmatic, randomised controlled trial at the Clinical Centre, University of Sarajevo between March 2018 and July 2020. The study was part of a larger Global Health Research Programme, funded by the National Institute of Health Research in the United Kingdom, which included studies on resource-oriented interventions in Bosnia and Herzegovina, Colombia and Uganda. Full study details are outlined in a published protocol, (Priebe *et al*., 2019) and the trial was prospectively registered with the ISRCTN registry (ISRCTN: 51290984). Ethical approval was obtained from the University of Sarajevo (18/09/2018) and Queen Mary University of London (ref: QMERC2018/66, 30/10/2018).

Patients were eligible to participate if they had: a primary diagnosis of schizophrenia or non-affective psychosis (ICD-10 F20-29), were aged 18 years and older, had capacity to provide informed consent; were experiencing poor quality of life (defined as scoring ⩽5 on the Manchester Short Assessment of Quality of Life (MANSA) scale) (Priebe *et al*., 1999), and had been diagnosed with schizophrenia for more than 6 months. Exclusion criteria included a primary diagnosis of substance-use disorder, learning disability, dementia, organic psychosis or bipolar disorder, being an inpatient at the time of recruitment and participating in another research study.

Patients were recruited from one site with an outpatient clinic and day hospital. Members of the clinical team screened patients with the support of researchers to identify potentially eligible patients, who were then approached by researchers and provided information about the study. Patients who provided written consent then completed screening with the MANSA to assess eligibility.

Volunteer befrienders were recruited through medical and psychology student organisations. To participate, volunteers were required to commit to meet regularly with patients over a 6-month period, attend training and supervision and be 18 years of age or older. Eligible individuals were provided information about the study by a researcher and invited to apply if interested. After an interview to confirm eligibility and determine their motivation for participation, interests and availability, volunteers provided written consent to participate.

Following recruitment, patients were randomly assigned to the intervention or treatment as usual group at a 1:1 ratio. Randomisation was conducted by an independent researcher using sequential computer-generated random numbers to determine allocation. Allocation information was provided to the unblinded research coordinator at the site who conducted baseline assessments.

The trial was pragmatic. The study design did not influence any treatment that the patients received during the study period other than the befriending programme in the intervention group.

### Intervention and procedure

A volunteer befriending intervention was developed following consultation with mental health professionals, service users, students and non-government organisations in Sarajevo. The intervention matched one volunteer befriender with one patient, and the pair agreed to meet every two weeks over the 6-month intervention period. The pair could decide what they would like to do at each meeting, including the option of meeting other pairs to take part in group social activities. During the intervention period, three group meetings were organised for pairs to join (an art class, a psychology workshop and a sports class). Pairs were matched based on their interests, preferences and availability, which was discussed during baseline assessments with patients and the recruitment interviews with potential volunteers. The volunteer coordinator then facilitated an initial meeting with each pair.

Before the intervention commenced, volunteers attended a one-day training course which covered general information about the programme, the symptoms of schizophrenia, the responsibilities and boundaries of befriending and available resources for supervision and support. They also received regular face-to-face supervision from a senior member of the research team and the volunteer coordinator, and three group supervision sessions were organised for the volunteers to share their experiences. The senior researcher was available to respond to any concerns that arose over the duration of the intervention. After 6 months, volunteers and patients could decide to continue meeting independently.

### Outcome measures

A standardised case report form was used to collect primary and secondary outcome data at baseline, 6 and 12 months. Socio-demographic information and clinical characteristics were assessed at baseline. Health service utilisation were measured at baseline and at each follow-up. Blinded researchers conducted outcome assessment at 6 and 12 months.

The primary outcome was subjective quality of life at 6 months, measured using the MANSA, which has been widely used in mental health research (Priebe *et al*., 1999). A mean score is calculated from patient satisfaction ratings of 12 life domains on a scale of 1 (‘couldn't be worse’) to 7 (‘couldn't be better’).

A range of secondary outcomes was measured. Mental health symptoms were assessed using the 24-item, observer-rated Brief Psychiatric Rating Scale (BPRS). (Lukoff *et al*., 1986) Each symptom was rated between 1 (‘not present’) and 7 (‘extremely severe’). All researchers were trained in BPRS assessments and inter-rater reliability was established before baseline data collection. The Objective Social Outcomes Index (SIX) (Priebe *et al*., 2008) was used to assess objective quality of life. Patients' employment, accommodation and social contacts are scored between 0 (poorest situation) to 6 (best situation). Two binary outcome items were adapted from existing instruments to measure friendships (Oliver *et al*., 1997; Priebe *et al*., 1999): one objective measure (‘In the last week, have you visited a friend, been visited by a friend or met a friend outside both your home and work?’) and one subjective measure (‘Do you have anyone who you would call a close friend?’).

Volunteers reported the number and lengths of meetings with patients to the coordinator via text message over the 12-month study period.

### Patient and public involvement

How to use volunteer befriending in Bosnia and Herzegovina was influenced by discussions with representatives of the patient organisations ‘Menssana’, ‘Dodir’ and ‘Fenix’. They welcomed the idea of the intervention and were also in support of a randomised controlled study design which is still unusual in the national context. They also supported recruitment of study participants, but were not involved in the conduct of the study. The organisations will receive a report with the findings of the study, once they have been published.

### Statistical analysis

All analysis was conducted in accordance with a pre-agreed statistical analysis plan by a blinded researcher. A sample size of 72 was agreed to ensure a minimum of 30 patients were enrolled in each arm (De Jong and Davidson, 1994; Browne, 1995) and to allow for 20% study attrition, which has been observed in previous research with people with psychosis (Szymczynska *et al*., 2017).

Baseline characteristics of both trial arms are presented descriptively. We use generalised mixed linear models (with a fixed effect for treatment and adjusted for baseline values) to compare mean MANSA and BPRS scores in each arm at baseline to 6- and 12-months and proportional odds models (with treatment as a fixed effect) to compare objective social outcomes (SIX). All analysis was conducted on available cases on an intention-to-treat basis. Cohen's *d* is presented as a standardised measure of effect.

## Results

### Participants

**Fig. 1.**
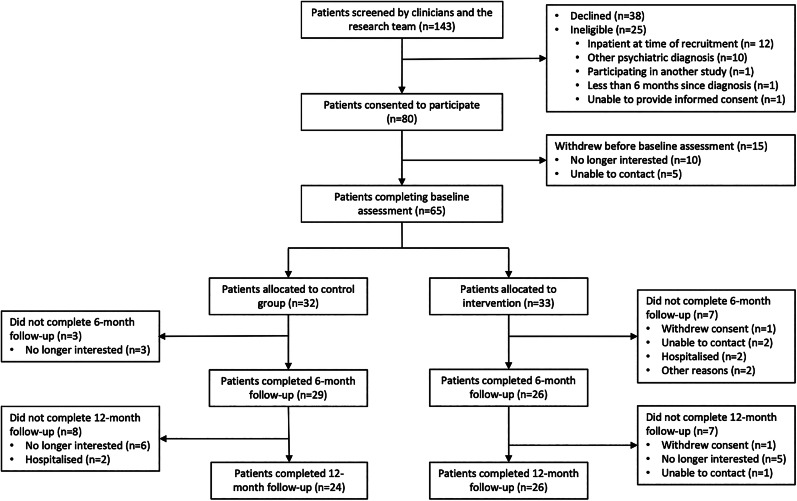
CONSORT patient recruitment flow diagram.

Of the 143 patient records screened for participation in the study, 118 met our eligibility criteria and 80 provided written consent to participate. The flow of patients through the study is summarised in Fig. 1.

During baseline assessment, five patients were unable to be contacted and 10 were no longer interested. The remaining 65 patients were randomised into the intervention (*n* = 33) and control arms (*n* = 32). Baseline characteristics of both groups are presented in Table 1.

**Table 1.** Patient characteristics at baseline

	Intervention (*n* = 33)	Control (*n* = 32)
Mean age in years (range)	42 (21–68)	44 (22–64)
Sex		
Male	14 (42%)	16 (50%)
Female	19 (58%)	16 (50%)
Marital status		
Single/unmarried	18 (55%)	16 (50%)
Married	7 (21%)	8 (25%)
Divorced/separated/widowed	8 (24%)	8 (25%)
Education		
Primary or less	4 (12%)	3 (9%)
Secondary	19 (58%)	25 (78%)
Tertiary or higher	10 (30%)	4 (13%)
Living with		
Family/partner	28 (85%)	29 (91%)
Alone	4 (12%)	2 (6%)
Friends/relatives	1 (3%)	1 (3%)
Employment		
Full time	6 (18%)	8 (25%)
Part time	2 (6%)	4 (13%)
Unemployed	11 (33%)	12 (38%)
On pension	6 (18%)	6 (19%)
Other	3 (9%)	2 (6%)
Physical comorbidities		
Cancer	1 (3%)	1 (3%)
Musculoskeletal problems	5 (15%)	1 (3%)
Diabetes	1 (3%)	2 (6%)
Cardiovascular disease	2 (6%)	4 (13%)
Hypertension	7 (21%)	7 (22%)
High cholesterol	8 (24%)	6 (19%)
Admitted to psychiatric in-patient treatment in the past 3 months	7 (21%)	9 (28%)
Seen by a psychologist in the past 3 months	10 (30%)	11 (34%)
Prescribed antipsychotic medication in the past 3 months	33 (100%)	29 (91%)

The mean age of participants was 43 (range: 21–68) and 54% were female. The two groups did not show substantial differences on any of the assessed socio-demographic and clinical characteristics.

After advertising the intervention, 110 students applied to participate as volunteers and interviews were scheduled with 75. After checking eligibility, 36 volunteers were chosen to participate, and another six recruited as reserves in the case of drop out. The mean age of participating volunteers was 25 (range 22–29) and 75% were female. All volunteers were either full time (64%) or part-time (36%) university students.

Patients in the intervention group were matched with one volunteer to create 33 pairs. Twenty-eight pairs attended an initial meeting facilitated by the study coordinator, while five pairs never met one another. The pairs met on average five times over the 6-month intervention period (range 0–12), and 25 pairs (75%) attended the pre-defined threshold of at least three out of the 12 recommended meetings over 6 months. After the initial meeting, one patient withdrew from the study and one patient was rematched to another volunteer based on patient preferences. Fifteen pairs (45%) continued to meet together after the intervention period had ended.

During the 6-month follow-up, four patients in the intervention group and two patients (3) in the control group were seen by a psychologist, and 28 patients (23) in the intervention group and 25 patients (22) in the control group were prescribed anti-psychotics. No patient in any of the two groups received further psycho-social treatments during the study period.

Because the pandemic imposed restrictions on social contacts, further face-to-face meetings between volunteers and patients following the 6-month intervention period were difficult or impossible, and 30% of 6-month interviews and all 12-month interviews had to be conducted via telephone or online. There were no other deviations from the protocol.

### Outcomes

At the end of the 6-month intervention period, 55 patients (intervention: *n* = 26, control: *n* = 29) completed follow-up assessments and at 12 months, 50 patients (intervention: *n* = 26, control: *n* = 24) completed an assessment. The results of outcome assessments are shown in Table 2.

**Table 2.** Outcomes of patients at baseline, 6 and 12 months (primary outcome: MANSA at 6 months)

	Intervention		Control		Coefficient (95% CI)*	*p*-value
	*n*	mean (SD)	*n*	mean (SD)		
MANSA						
Baseline	33	4.4 ± 0.6	32	4.3 ± 0.6	…	…
6 months	29	5.1 ± 0.8	26	4.3 ± 1.0	−0.50 (−0.94 − −0.07)	0.024
12 months	26	5.7 ± 0.7	24	3.9 ± 1.2	−1.52 (−2.37 − −0.67)	0.001
SIX						
Baseline	33	3.9 ± 1.2	32	4.1 ± 1.3	…	…
6 months	29	4.2 ± 1.2	26	4.2 ± 1.5	1.48 (−0.13–3.08)	0.072
12 months	26	4.8 ± 1.3	24	3.6 ± 1.4	2.20 (0.50–3.90)	0.011
BPRS						
Baseline	33	33.8 ± 7.1	32	35.6 ± 7.1	…	…
6 months	29	29.6 ± 3.7	26	36.8 ± 9.8	5.29 (1.94–8.64)	0.003
12 months	26	30.7 ± 4.5	24	37.7 ± 5.5	6.85 (3.02–10.67)	0.001

*For the MANSA and BPRS, regression coefficients derived from mixed linear models with a fixed effect for treatment adjusted for baseline scores. For the SIX, proportional odds models with a random intercept and fixed effect for treatment.

After 6 months, there was no difference between participants and controls in the proportion reporting having someone they would call a close friend, or the proportion having seen a friend in the past week. However, at 12-month assessment participants were more likely to have someone they would call a close friend (92%) compared with controls (54%, *p* = 0.003). Similarly, they were more likely to have seen a friend in the past week (participants: 85%, controls: 29%, *p* < 0.001).

Primary and secondary outcome measurements at all three time points are presented in Table 2. The intervention had a significant effect on our primary outcome, quality of life at 6 months (Cohen's *d* = 0.88, *p* = 0.024,) and at quality of life at 12 months (*d* = 1.83, *p* = 0.001). Significant effects were also observed in psychiatric symptoms at 6 months (*d* = 0.97, *p* = 0.003) and 12 months (*d* = 1.39, *p* = 0.001). For objective social outcomes (SIX), there was a trend towards better scores at 6 months (proportional odds ratio = 1.48 [95% CI −0.13–3.08, *p* = 0.072]) and significant effect at 12 months (proportional odds ratio = 2.20 [95% CI 0.50–3.90, *p* = 0.011]).

In a sensitivity analysis, we repeated the analysis of the primary outcome of quality of life at 6 months with imputed data for missing patients, using multiple imputation by fully conditional specification. This did not alter the finding of the analysis of available cases as reported before (*d* = 0.91, *p* = 0.001).

### Patient and volunteer experiences

After the 6-month intervention period, 15 patients and 15 volunteers were interviewed about their experiences and some of their accounts are presented in Table 3.

**Table 3.** Patient and volunteer accounts of participating in the intervention

Patient experiences
‘*Before the programme I was worried how it was all going to look like. Are we going to get along? What would we talk about? Who would the person be? I was wondering, why am I doing all this? But now I'm happy I did it. …Afterwards, it was easier for me to contact and talk to new people and even people I already know. I started to hang out more with other people, that's something I wasn't used to…* ' (Patient 010)
‘*…in the beginning I was very sceptical, but as it started developing, I changed my mind and at this point I can say it is a great thing for people with mental illness… I think it is so important to have an intervention like this because I think it is important to have something more than just medication… you just need something beside the drugs.*’ (Patient 051)
‘*Having someone to go out with beside my mum, was meaningful for me, especially just after the hospitalisation… my family was with me of course, providing support but having someone who is not from your house is very different. I believe I have one more friend now. I'm glad we were introduced to each other and matched this way. We've continued contact and meetings after the intervention too.*' (Patient 058)
Volunteer experiences
*Throughout our friendship, I felt like his self-confidence raised, he became to start the conversation, also to invite other people out, and that also made me feel happy and somehow, at least partly, responsible for that. That rewarding feeling was something that made me to go to the next meeting… I couldn't imagine a better feeling than helping that young man feel better about himself.*' (Volunteer 032)
*I noticed that he was a bit different, a bit more open every time we met. He started to talk more about going out with people… I believe that's what motivated me the most to continue with meetings.*’ (Volunteer 025)
‘*I've never had contact with someone who has severe mental illness, but I didn't expect it to be this way. I expected to have much more difficulties in communication, so I was truly surprised when my friend and I started to talk about things easily, even some things I didn't used to share easily with some of my closest people.'* (Volunteer 004)
‘*From this perspective, all those stereotypes about mentally ill people seem so unnecessary and exaggerated. I've never regretted being a part of this programme, and something similar ever happens again, I would be happy to be a part of it*.’ (Volunteer 032)

## Discussion

This is the first randomised controlled trial of volunteer befriending for patients with schizophrenia in Bosnia and Herzegovina and one of the first conducted anywhere in the world (Harris *et al*., 1999; Davidson *et al*., 2004; Sheridan *et al*., 2015; Priebe *et al*., 2020). We observed large and sustained improvements in both objective and subjective measures of quality of life and reductions in psychiatric symptoms. Patients in the intervention group had more social contacts with friends and were more likely to have a close friend at 12-month follow-up, compared with controls. The intervention appears feasible and acceptable in this context. Pairs met regularly over the intervention period and 75% attended our pre-defined minimum number of meetings. We had lower than expected attrition from the study and 45% of pairs chose to stay in contact after the intervention period ended. These findings were illustrated by positive accounts by both patients and volunteers.

Patients in the intervention group had, as compared to the control group, a more favourable change in the mean MANSA score of 0.7 points at 6 months and of 1.7 points at 12 months. This is a clinically highly relevant benefit. An improvement of 0.7 points is equivalent to patients rating their satisfaction with at least eight of 12 life areas more positively by at least one of seven scale points. An improvement of 1.7 points is equivalent to patients rating on average all life domains more than one scale point more positively. The effect sizes are unusually high for psycho-social intervention with patients with psychotic disorders.

### Strengths and limitations

The intervention was developed through consultation with local stakeholders (patients, clinicians and non-government organisations) and training sessions, supervision and support was provided to all volunteers. Valid and reliable outcome measures were used and data collected at multiple time points. These measures including a mix of both self-rated and observer-rated scales, and significant positive effects were found on both self-ratings and observer-ratings.

There are also some limitations in this study. The trial was conducted at only one site, which may affect the generalisability. Loss to follow-up at 6 months was 15% and 23% at 12 month, which may introduce some bias. However, this is below average for RCTs of non-pharmacological studies in people with severe mental illness (Szymczynska *et al*., 2017). Restrictions introduced due to the coronavirus disease 2019 pandemic interrupted data collection activities. At 6 months, 70% of follow-up assessments had been completed when restrictions were introduced. The remaining assessments, and all 12-month assessments, were conducted via telephone rather than face to face, which may have introduced an additional variance. This was the first psychosocial intervention trialled at the University of Sarajevo Clinical Centre. As research is not very common in Bosnia and Herzegovina and patients were unfamiliar with trial and randomisation processes, some screened patients who initially showed interest in the study were later not willing to participate. As a result, the target sample size of 72 patients was not achieved. Finally, as volunteers were predominantly recruited through advertisements at universities, they were generally younger (mean age: 25) than the patients they befriended (mean age: 43). This, together with the fact our volunteers were predominantly female, may limit the generalisability of our findings.

### Comparison with literature

While there have been few studies examining volunteer befriending in other low- and middle-income countries, a group befriending intervention in Colombia for people with severe mental illness was found to be feasible and acceptable (Botero-Rodríguez *et al*.). Globally, peer support interventions, in which patients are matched with volunteers with lived experience of mental illness, are attracting increased attention (Ryan *et al*., 2019) and these programmes have been shown to be sustainable in low-resource settings (Atif *et al*., 2019).

Previous studies of befriending interventions for people with severe mental illness in high-income countries have found a positive effect of these programmes on social isolation and social functioning (McCorkle *et al*., 2008; Sheridan *et al*., 2015; Priebe *et al*., 2020). However, these studies found no subsequent improvements in quality of life or depressive symptoms. In Bosnia and Herzegovina, treatment as usual for patients with schizophrenia primarily consists of routine meetings with clinicians and pharmacotherapy, and rarely includes psychosocial support (Winkler *et al*., 2017). The large improvements in quality of life and symptom severity observed in our study may, in part, reflect the absence of a comprehensive package of usual care (Asher *et al*., 2017).

Evidence from other settings has shown that while most people with severe mental illness are willing to participate in befriending interventions, there is considerable variability in their preferences for delivery (Toner *et al*., 2018*a*). The improvements observed by participants may also reflect our decision to design an intervention which was flexibly delivered, in terms of the content, frequency and duration of meetings and activities.

Reviews of befriending programmes through volunteers in mental health show a large variation of personal characteristics of volunteers, often reflecting specific aspects of the local context and the set-up of the given programme (Hallett *et al*., 2012; Toner *et al*., 2018*b*). Like in a similar trial in London (Priebe *et al*., 2020), many volunteers in this study were young students. They had varied motivations and expectations from participating, but many hoped to gain experience and skills that might advance their future careers in psychology and medicine. Such motivation is frequent in volunteering programmes and has been described in more detail in qualitative studies (Cassidy *et al*., 2019). It is however different from the main motivation of volunteers in many other programmes, where volunteers are often older than the patients (Klug *et al*., 2018), and may influence feasibility, processes and outcomes of programmes.

### Implications for research and practice

Volunteer befriending is a low-cost intervention, which appears feasible, acceptable and effective for patients with schizophrenia in Bosnia and Herzegovina, a middle-income country. Qualitative data show the intervention was valued by both patients and participants. This suggests volunteer befriending could be delivered and implemented in these settings. Future research is required to better understand implementation processes and explore the characteristics and motivations of volunteers and patients, in order to support the implementation and potential sustainability of these programmes in the longer term. This information may inform the wider adoption and implementation of volunteer befriending programmes in other low-resource settings.

## Data Availability

Anonymised data arising from the study will be available to external researchers upon reasonable request based on a signed data-sharing agreement.
